# Facilitators and Barriers of Relatives' Involvement in Care of Patients With Acquired Brain Injury or Malignant Brain Tumour: Scoping Review

**DOI:** 10.1002/nop2.70417

**Published:** 2026-01-20

**Authors:** Rikke Guldager, Pernille Sejr Smedegaard, Sara Nordentoft, Lena Aadal, Mia Ingerslev Loft, Ingrid Poulsen

**Affiliations:** ^1^ Department of Neurosurgery Copenhagen University Hospital – Rigshospitalet Copenhagen Denmark; ^2^ Department of People and Technology Roskilde University Roskilde Denmark; ^3^ Hammel Neurorehabilitation and Research Centre Hammel Denmark; ^4^ Department of Clinical Medicine Aarhus Denmark; ^5^ Department of Neurology Copenhagen University Hospital – Rigshospitalet Copenhagen Denmark; ^6^ Research Unit Nursing and Health Care Aarhus University Aarhus Denmark; ^7^ Department of Clinical Research Copenhagen University Hospital ‐ Amager and Hvidovre Hospital Hvidovre Denmark

**Keywords:** acquired brain injury, involvement, malignant brain tumour, nurses, relatives

## Abstract

**Aim:**

To identify and map the breadth of available evidence on nurses' perspectives of the facilitators and barriers to relatives' involvement in the care continuum of patients with acquired brain injury or malignant brain tumour.

**Background:**

The involvement of relatives in care and treatment may have a significant positive impact on the quality of care and treatment, leading to higher satisfaction with hospitalisation for patients, relatives and healthcare professionals. Nurses play an important role in nurturing a trusting and facilitating relatives' involvement. However, involving relatives seems complex and multifaceted, with many possible facilitators and barriers to consider.

**Design:**

This scoping review was conducted in accordance with the Joanna Briggs Institute's methodology for scoping reviews and a published a priori protocol.

**Data Sources:**

A comprehensive literature search was conducted in MEDLINE (PubMed), CINAHL (EBSCO) and Embase (OVID). Reference lists of included studies, Google Scholar and Web of Science were also searched. Literature published in the English, German or Scandinavian languages since 2010 was included.

**Results:**

The search identified 4330 studies, of which 18 were included. No studies including involvement of relatives to patients with Malignant Brain Tumour was found. Nurses' perspectives of the facilitators and barriers to relatives' involvement of patients with acquired brain injury indicates that several facilitators and barriers contribute to or hinder relatives' involvement. The facilitators for involvement were mostly related to nursing tasks within the healthcare system, acknowledging relatives in their own rights, building a trusting relationship and using communication as a tool. Contrary, barriers were organisational factors, when the patient was seen as a primary focus of care, and informational challenges.

**Conclusions:**

The results illustrate the complex nature of involvement from the perspective of nurses. The results indicate a paradox because several of the identified aspects are not mutually exclusive but rather represent aspects of involvement that range along a continuum.

**Implication for the Profession:**

Nurses' involvement of relatives in the care continuum is important, however evidence suggests that the relationships between relatives and nurses need to be strengthened to individualise the level of involvement. We suggest that the organisational and contextual factors that shape relative involvement need to be studied further.

**Report Method:**

PRISMA‐ScR.

**Patient or Public Contribution:**

No Patient or Public Contribution. However, the review findings were shared and discussed with a panel of nurses from the neurosurgical speciality who validated and nuanced the findings into a Danish context.

## 1 Introduction

Acquired brain injury (ABI) and malignant brain tumour (MBT) are serious conditions that profoundly affect patients' cognitive, physical, social and psychological well‐being. The symptoms linked to both ABI and MBT encompass physical manifestations such as aphasia, hemiparesis, fatigue, apathy and seizures, as well as psychosocial symptoms like anxiety, stress and depression. Additionally, cognitive symptoms occur including concentration problems, personality changes, reduced attention and short‐term memory impairments (Molassiotis et al. 2010; Mar et al. 2011; Piil et al. 2022).

An ABI encompasses both traumatic and nontraumatic brain injuries that occur postnatally (Goldman et al. 2022). Traumatic brain injuries (TBI) are typically a result of falls, roads or sports accidents, resulting in hematoma or concussions (Ahmed et al. 2017). Nontraumatic brain injuries include conditions such as stroke and aneurysms. On a global scale, approximately 13.7 million new stroke cases emerge each year, resulting in over 116 million years of healthy life being lost because of stroke‐related deaths and disabilities (George 2020). In the United States and Europe, the estimated incidence is around 30 cases per 100,000 persons per year (Piil et al. 2022). MBT is characterised as a rapidly progressive, life‐threatening disease primarily affecting individuals aged 45 to 70 years (Ostrom et al. 2021, 2014). Patients diagnosed with MBT often face a poor prognosis, as the symptoms tend to develop rapidly as the disease progresses (Ostrom et al. 2021).

Suffering from an ABI or MBT can be profoundly devastating and life‐altering for both patients and their relatives (Guldager et al. 2023; Guldager, Nordentoft, et al. 2022). The sudden onset of these diseases inflicts intense emotional distress on both patients and their relatives where they, besides being in acute crisis, also struggle to adjust to the changed family dynamics (Fisher et al. 2019). Over time, relatives often find themselves assuming new caretaking responsibilities for the patient, which may lead to the patient depending more on their relatives' support and advocacy (Guldager et al. 2023; Guldager, Nordentoft, et al. 2022). Moreover, cognitive impairments may hinder the patients' ability effectively collaborate with healthcare professionals (HCPs) and participate in decision‐making of treatment and care (Mar et al. 2011). Meeting patients' and relatives' need for being involved in decision‐making processes regarding their treatment and care may be challenging because a lack of education on how to conduct therapeutic conversations with relatives and lack of time can act as barriers to nurses (Vestala and Frisman 2013; Kwame and Petrucka 2021; Cranley et al. 2022). Furthermore, an organisational and work environment that is not conducive may negatively influence involvement of both patient and relative (Kwame and Petrucka 2021; Keatinge et al. 2002). Research conducted by (Guldager et al. 2019) illustrated that differential and unequal resources among relatives serve as both facilitators and barriers to patient involvement, particular among patients with TBI (Guldager et al. 2019). The facilitators for involvement included being participating in nursing care situations, including the possibility of being present during hospitalisation, a relative's relationship with the HCPs, experience with illness, dedication and proactivity in the decision‐making process and in nursing care situations. Conversely, barriers to involvement encompass being reactive, nonparticipating in nursing care situations, unable to express one's own wants and needs and limited flexibility from workplace (Guldager et al. 2019).

Achieving successful involvement of relatives necessitates the recognition of distinct areas of expertise between understanding relatives and the specialised knowledge held by nurses, each operating within their respective spheres of expertise. Nurses are experts in providing nursing care and the quality of nursing care relies on a series of actions mediated through an emphatic relationship built by the nurses (Kitson et al. 2013). On the other hand, relatives, in many instances, possess valuable person‐specific knowledge that nurses may not have access to (Gillespie 2010; Gillespie and Peterson 2009).

Despite the research demonstrating the positive effects of involving relatives in patient care, such as improved treatment outcomes (Videnscenter for Brugerinddragelse i Sundhedsvæsenet 2015); enhanced psychological and emotional well‐being of patients (Dansk Selskab for Patientsikkerhed 2014); and increased safety in patient care (Baines and Bere 2018), there are many facilitators and barriers to involvement. The responsibility for facilitating the involvement of relatives is a shared task among various healthcare professions, with nurses playing an essential role in this regard (Cranley et al. 2022). Several studies have investigated specific interventions facilitating the involvement of relatives of patients with an ABI or a MBT (Araújo et al. 2018; Avci and Gozum 2021; Halkett et al. 2023). However, the evidence specifically on nurses' facilitators and barriers to involvement of relatives of patients with an ABI or a MBT has not previously been mapped. This could be explained by the fact that little attention has been given to the conceptual meaning of involvement (Thompson 2007; Lundh et al. 2024). Involvement, shared decision making and collaboration are all concepts closely related and frequently used interchangeably (Lundh et al. 2024; Vahdat et al. 2014). Identifying and mapping the available evidence of the facilitators and barriers from the perspective of the nurses in studies where only nurses participated and nurses as a part of multidisciplinary team could form a background for the development of education programmes or interventions that facilitate collaboration and partnerships in care, implementations of policies or organisational changes to involve relatives in treatment and care.

## 2 The Review

### 2.1 Aim

The objective of this scoping review is to identify and map the breadth of available evidence on the possible facilitators and barriers to nurses' involvement of relatives of patients with ABI and MBT on the care continuum.

### 2.2 Design

This review followed the JBI methodology for scoping reviews (Peters, Marnie, Tricco, et al. 2020; Peters, Godfrey, McInerney, et al. 2020) and was conducted according to an a priori protocol (Guldager, Loft, et al. 2022). The review is reported in accordance with the PRISMA extension for scoping reviews (Tricco et al. 2018).

#### 2.2.1 Deviations From the Protocol

We previously published a scoping review protocol titled ‘Facilitators and barriers of relatives' involvement in nursing care decisions and self‐care of patients with ABI or malignant brain tumour: a scoping review protocol’ (Guldager, Loft, et al. 2022). However, we have decided to make deviations from the protocol since we wanted to explore involvement of relatives in general, meaning in all interactions between nurses and relatives and not only in nursing care decisions or selfcare. These adjustments have been reflected throughout the manuscript.

### 2.3 Search Methods

A preliminary search of Medline (via PubMed), the Cochrane Database of Systematic Reviews and *JBI Evidence Synthesis* was conducted in September 2021, and no current or in‐progress systematic reviews or scoping reviews on the topic were identified.

Our search strategy aimed to identify both published and unpublished studies (grey literature). A three‐step search strategy was utilised as recommended by JBI (Tricco et al. 2018). The first step consisted of an initial preliminary search of MEDLINE (PubMed), CINAHL (EBSCO) and Embase (OVID) for the possible facilitators and barriers from the perspective of nurses involving the relatives of patients with ABI throughout the care continuum. The search for grey literature was conducted in Web of Science and Google Scholar, in accordance with the methods described by Bramer et al. (2016). The literature search was followed by an analysis of the text contained in both the titles and abstracts of the retrieved studies and of the key‐ and index terms used to describe the articles. This informed the development of a search strategy, including an identification of the search terms and index terms tailored for each database. Key terms for the search strategy were developed by the two of the authors (RG and IP) in collaboration with an information specialist. The full search strategy is provided in Appendix S1. The reference lists of all included studies were screened for additional studies. Only studies in English, German or Scandinavian languages were included for pragmatic reasons. An extensive search was performed in December 2021: updated and rerun in October 2022 and December 2023. A date filter of 2010–2023 was selected because of the increased focus the involvement on relatives during this period, attributed to factors such as a growing elderly population, the implementation of fast‐track programmes, and shortened hospital stays (Norlyk and Martinsen 2013). These factors have led to reduction in time and contact with HCPs and consequently an increase in extra caregiving responsibilities for relatives, who occupy a dual role that encompasses both the support typically provided by HCPs and that of a caring family member (Guldager et al. 2023; Guldager, Nordentoft, et al. 2022).

### 2.4 Inclusion and Exclusion Criteria

Studies which included data from participants who met the following inclusion criteria: nurse's involvement of relatives (≥ 18 years) to patients (≥ 18 years) with an ABI or MBT in all settings. In this scoping review, a relative is defined as an individual who provides informal care or as a representative for the patient based on their knowledge given normal preferences (Steenfeldt 2004). Informal care is also characterised as unpaid assistance given to dependent individuals by individuals who share a social connection, including spouses, parents, children, other relatives, neighbours, friends or other non‐kin individuals (Triantafillou et al. 2010).

The overarching concept of the scoping review was to comprehensively examine the possible facilitators and barriers from the perspective of nurses' when involving the relatives of patients with ABI or MBT in various settings. Our objective was to systematically identify the relevant literature that describes both the facilitators and barriers independently, as well as any potential relationship between them. This review encompasses studies that provided insights into the perspectives of nurses' when it comes to the involvement of relatives of patients with ABI or MBT in their nursing care. Studies concentrating on patients with neurogenerative diseases, such as Parkinson's, Alzheimer's, dementia or multiple sclerosis, were excluded. The scoping review had no context restrictions.

### 2.5 Study Selection

The database search results were imported into the electronic bibliographic database Endnote v.20 (Clarivate Analytics PA, USA) and then collated and uploaded into Covidence (Veritas Health Innovation, Melbourne, Australia), which automatically removed the duplicates. The selection of the studies published in the electronic databases was conducted in two stages. First, two authors (RG and PSS) screened the titles and abstracts against the predetermined inclusion criteria. Conflicts at this stage were resolved through a discussion. Second, full‐text records were assessed for final inclusion. Disagreements were resolved through discussion and if consensus could not be reached a third author was consulted (SN). However, this opportunity was not used. Additional studies were identified by checking the reference lists of the included studies, and these were screened using the same approach. A list of the studies during the full‐text screening is reported along with our reasons for exclusion in Appendix S2. Consistent with the JBI methodology for scoping reviews, a formal appraisal of included papers was not performed (Peters, Godfrey, McInerney, et al. 2020).

### 2.6 Quality Appraisal

A formal appraisal of the methodological quality of included studies is generally not performed in scoping reviews (Peters, Godfrey, Mclnerney, et al. 2020) and was therefore not done.

### 2.7 Data Extraction and Mapping of Results

A data extraction tool was developed a priori, as per the JBI methodology (Peters, Marnie, Tricco, et al. 2020; Peters, Godfrey, McInerney, et al. 2020; Guldager, Loft, et al. 2022), to ensure that the relevant data of the selected studies were extracted (Appendix S3). The data extraction tool was trialled on three studies to obtain adequately detailed information on the included studies. The rest of the research team checked for completeness and accuracy of the extracted data, especially those related to classifying a facilitator or barrier. The first two authors independently extracted data from the included studies using the data extraction tool. This enabled a logical and descriptive summary of the findings that aligned with the objective of the review. Information on key characteristics of study's authors, year of publication, origin/country of origin, setting, population and methodology/methods. Findings related to the perspective of nurses in studies where only nurses participated and nurses as a part of multidisciplinary team on the involvement of relatives was extracted. An ongoing cross‐check from five random samples of the included studies was conducted by two authors to ensure the accuracy of the extracted data. Disagreements were resolved by consensus or consultation with a third author. The full abstraction tables can be accessed upon request.

As recommended by the *JBI* methodology (Peters, Marnie, Tricco, et al. 2020; Peters, Godfrey, McInerney, et al. 2020), textual data mapping the facilitators and barriers to involvement were synthesised narratively using a descriptive qualitative content analysis that helped in minimising interpretation (Peters, Marnie, Tricco, et al. 2020; Peters, Godfrey, McInerney, et al. 2020). Data were extracted directly and included verbatim information regarding the facilitators and barriers to involvement. A descriptive summary of the review's findings is presented with tables and figures to support the data, where appropriate.

### 2.8 Nurses Involvement in Research (PPI)

Nurses were involved to validate, refine and adapt the review findings for application within a national context, ensuring their relevance to (Danish) nurses. To guide the PPI process, ‘A Researcher's Guide to Patient and Public Involvement – a guide based on the experiences of health and medical researchers, patients, and members of the public’ was employed (Turk et al. 2017; Locock et al. 2017; Crocker et al. 2017). After the scoping review was completed, a panel of five nurses from the neurosurgery department was convened. The panel did not participate in conducting the scoping review, nor did they influence its aim or scope. By reflecting on the results, the nurses were able to identify potential barriers and challenges, that might arise when implementing the involvement of relatives in practice.

### 2.9 Research Ethics

This scoping review does not require ethical approval, as it involves the analysis of publicly available data. The findings will be disseminated through peer‐reviewed publications, conference presentations and knowledge mobilisation activities.

## 3 Results

### 3.1 Study Inclusion

In total 5682 papers were identified in our database search, and 1165 duplicates were removed. By reading titles and abstracts 4460 papers were excluded. Of these papers, 57 papers underwent full‐text review by the same two authors (RG and PSSD), resulting in the inclusion of 18 studies. Thirty‐nine studies did not meet the inclusion criteria and were excluded (Appendix S2).

**FIGURE 1:**
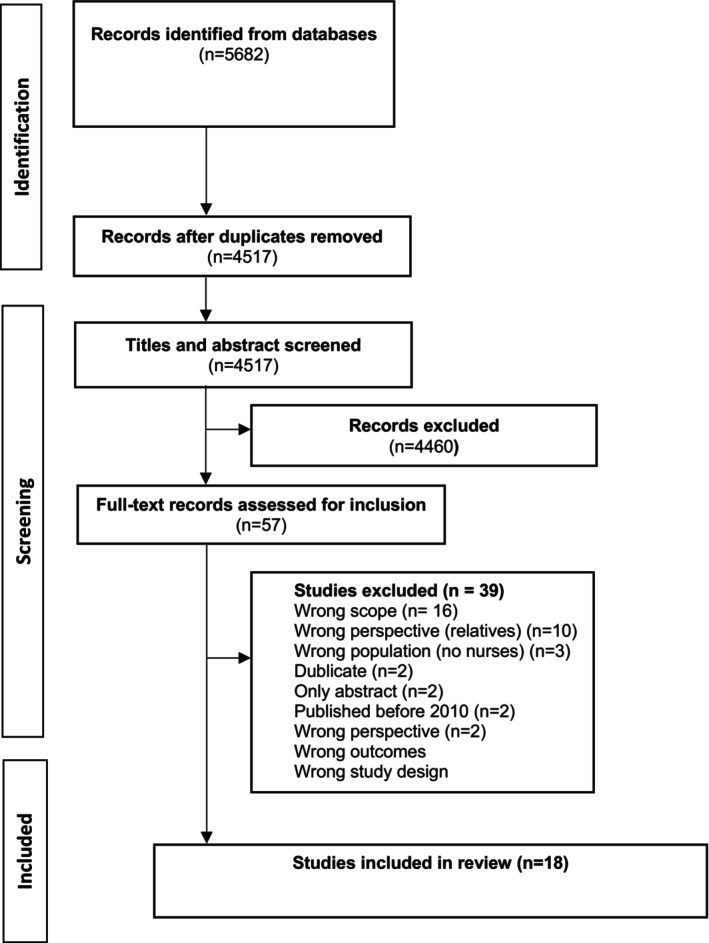
Flow diagram of study inclusion.

In total, 18 studies were identified for inclusion. The PRISMA flow diagram (see Figure 1) outlines the process for the inclusion of studies.

### 3.2 Characteristics of the Included Studies

The aim of this scoping review was to identify and map the breadth of available evidence on nurses' perspectives on the facilitators and barriers to relatives' involvement in the care continuum of patients with ABI and MBT. Unfortunately, no papers on the involvement of relatives to the MBT population met the inclusion criteria, and all 18 studies included represent the population of patients diagnosed with ABI (Table 1). However, it was not possible to extract data solely from the perspectives of nurses, as their collaborative work typically occurs within a multidisciplinary team. A presentation of the key characteristics of the included studies is summarised in detail in Appendix S4.

**TABLE 1:** Characteristics of the 18 included studies.

Study characteristics	Numbers of studies (%)^a^	
Year of publication	2012	1 (6)
	2013	1 (6)
	2014	2 (11)
	2015	2 (11)
	2016	2 (11)
	2017	2 (11)
	2018	1 (6)
	2019	3 (17)
	2020	3 (17)
	2021	1 (6)
Continent of publication	Europe	9 (50)
	North America	6 (33)
	Australia	2 (11)
	Asia	1 (6)
Study population	Nurses	161 (44)
	Other health care professionals^b^	203 (56)
Biological relation to the patient	Spouses	71 (35)
	Parents	35 (17)
	Adult children	33 (16)
	Siblings	5 (2)
	Other family	3 (1)
	Other	5 (2)
	Not reported	5 (2)
Diagnosis	Stroke	15 (83)
	TBI	2 (11)
	ABI	1 (6)
Settings	Acute care & rehabilitation	10 (56)
	Acute care	2 (11)
	Rehabilitation	1 (6)
	In‐hospital	3 (17)
	Community‐based	1 (6)
	Not reported	1 (6)
Methods	Qualitative	16 (89)
	Mixed methods	2 (11)
Study designs	Individual interviews	6 (33)
	Focus group interviews	5 (28)
	Individual interviews + focus group interviews	4 (22)
	Individual interviews, participant observation, document analysis	1 (6)
	Interview + survey	2 (11)

^a^ Percentages do not always add up to 100 due to rounding.

^b^ Doctors, health care assistants, physiotherapists, occupational therapists, speech and language therapists, psychologists, general practitioners, health‐ service managers and nurse‐assistants.

#### 3.2.1 Publication Date and Country of Origin

The 18 studies encompass data from the following countries: Canada (Cameron et al. 2014, 2013; Rochette et al. 2014; Lefebvre and Levert 2012), Denmark (Dreyer et al. 2016; Aadal et al. 2018), UK (Bennett 2017; Kyle et al. 2020; Al‐Janabi et al. 2019; Clancy et al. 2020), Australia (Kable et al. 2019), Finland (Lehto et al. 2019), Greece (Theofanidis and Gibbon 2016), Singapore (Ramazanu et al. 2020), USA (Hoewing 2017; Schutz et al. 2017), Norway (Solli et al. 2015) and New Zealand (Roy et al. 2015).

The studies were published between 2012 and 2021, with a majority of publications from 2019 (Al‐Janabi et al. 2019; Kable et al. 2019; Lehto et al. 2019) and 2020 (Kyle et al. 2020; Clancy et al. 2020; Ramazanu et al. 2020).

#### 3.2.2 Study Population and Sample Size

Altogether, the studies included nurses (*n* = 161) from a wide range of healthcare settings involved in care for patients with ABI. Apart from nurses, other HCPs were represented, comprising doctors, healthcare assistants, physiotherapists, occupational therapists, speech and language therapists, psychologists, general practitioners, health service managers and nurse‐assistants (*n* = 203). We considered a study for inclusion if the study population included the perspective of nurses' or nurses as a part of multidisciplinary team on the involvement of relatives, and if we could extract data reflecting their perspectives. Consequently, our focus was on findings that examined the perspectives of nurses and nurses working within a multidisciplinary team on the involvement of relatives.

The studies referred to relatives as family members (Bennett 2017; Lehto et al. 2019; Hoewing 2017; Schutz et al. 2017; Roy et al. 2015), caregivers (Cameron et al. 2014; Solli et al. 2015), family carers (Al‐Janabi et al. 2019), carers (Clancy et al. 2020), family caregivers (Cameron et al. 2013), loved ones (Lefebvre and Levert 2012) and spousal caregivers (Ramazanu et al. 2020). In 12 studies, relatives were a part of the population samples, where spousal caregivers constituted the greatest majority (*n* = 71), followed by parents (*n* = 35) and children (*n* = 33). Siblings (*n* = 5), other family members (*n* = 5) and others (*n* = 3) were also represented, whereas five studies (*n* = 5) did not report the relation.

#### 3.2.3 Patient Diagnosis

In the 18 included studies, the authors referred to the patient diagnosis as stroke (Cameron et al. 2014, 2013; Rochette et al. 2014; Dreyer et al. 2016; Aadal et al. 2018; Bennett 2017; Kyle et al. 2020; Al‐Janabi et al. 2019; Clancy et al. 2020; Kable et al. 2019; Lehto et al. 2019; Theofanidis and Gibbon 2016; Ramazanu et al. 2020; Solli et al. 2015; Roy et al. 2015), TBI (Lefebvre and Levert 2012; Hoewing 2017) or severe ABI (Schutz et al. 2017).

#### 3.2.4 Setting

The research was conducted across a variety of settings. Here, 10 of the 18 studies were conducted in a combination of acute care and the rehabilitation phase (Cameron et al. 2013; Rochette et al. 2014; Lefebvre and Levert 2012; Dreyer et al. 2016; Aadal et al. 2018; Bennett 2017; Kyle et al. 2020; Lehto et al. 2019; Theofanidis and Gibbon 2016; Roy et al. 2015), three studies were conducted in‐hospital (Cameron et al. 2014; Clancy et al. 2020; Kable et al. 2019), two exclusively in an acute care setting (Hoewing 2017; Schutz et al. 2017) and one in rehabilitation (Ramazanu et al. 2020). Only one study was in a community setting (Solli et al. 2015), and one study did not provide information about the setting in which it was conducted (Al‐Janabi et al. 2019).

#### 3.2.5 Study Design

Sixteen studies applied a qualitative study design (Cameron et al. 2014, 2013; Rochette et al. 2014; Lefebvre and Levert 2012; Dreyer et al. 2016; Aadal et al. 2018; Bennett 2017; Kyle et al. 2020; Al‐Janabi et al. 2019; Clancy et al. 2020; Kable et al. 2019; Lehto et al. 2019; Theofanidis and Gibbon 2016; Ramazanu et al. 2020; Schutz et al. 2017; Solli et al. 2015), including descriptive, interpretive, ethnographic and grounded theory approaches. Of the six studies that used individual interviews (Cameron et al. 2013; Kyle et al. 2020; Theofanidis and Gibbon 2016; Ramazanu et al. 2020; Schutz et al. 2017; Solli et al. 2015), five studies used focus groups (Lefebvre and Levert 2012; Dreyer et al. 2016; Aadal et al. 2018; Kable et al. 2019; Lehto et al. 2019), with four studies utilising both interviews and focus groups (Cameron et al. 2014; Rochette et al. 2014; Al‐Janabi et al. 2019; Clancy et al. 2020). One study applied a multimethod design using interviews, participant observation and document review (Bennett 2017). The remaining two studies applied a mixed methods study design using interviews and surveys (Hoewing 2017; Roy et al. 2015).

### 3.3 Review Findings

Based on the JBI scoping review guidelines (Peters, Marnie, Tricco, et al. 2020; Peters, Godfrey, McInerney, et al. 2020), the present study's results are reported narratively as a descriptive summary. Table 2 summarises the key facilitators and barriers identified across the included studies.

**TABLE 2:** Key characteristics for facilitators and barriers.

Author, year of publication	Nursing task within the healthcare system	Acknowledge relatives in their own right	Building a trusting relationship	Communication as a tool	Organisational factors of the healthcare system	Patient as primary focus of care	Informational challenges
	Facilitators for involvement				Barriers for involvement		
Al‐Janabi, 2019	x			x	x		
Bennett, 2017	x	x	x				x
Cameron, 2013	x	x			x	x	x
Cameron, 2014	x				x		x
Clancy, 2020					x		
Dreyer, 2016	x	x	x		x	x	
Hoewing, 2021					x	x	x
Kable, 2019				x	x		x
Kyle, 2020	x				x		x
Lefebre, 2012	x	x	x	x			x
Letho, 2019	x	x	x	x			
Ramazanu, 2020		x			x		x
Rochette, 2014					x	x	
Roy, 2015	x	x	x	x	x	x	x
Schutz, 2017			x	x			
Solli, 2015		x	x	x	x		
Theofandis, 2016					x		
Aadal, 2018	x	x		x	x	x	x
Total in studies	*n* = 10	*n* = 9	*n* = 7	*n* = 8	*n* = 14	*n* = 6	*n* = 10

### 3.4 Facilitators for Involving Relatives From the Nurses' Perspective

The most frequently occurring facilitator from the perspective of the nurses referred to different nursing tasks within the healthcare system. A total of 10 studies mentioned the task of supporting relatives to navigate in the healthcare system as a facilitator for involvement (Cameron et al. 2014, 2013; Lefebvre and Levert 2012; Dreyer et al. 2016; Aadal et al. 2018; Bennett 2017; Kyle et al. 2020; Al‐Janabi et al. 2019; Lehto et al. 2019; Ramazanu et al. 2020). This comprised supporting the relatives to figure out ‘how the system works’ and ease the journey through the patient pathway because the healthcare system was viewed by the relatives as an opaque institution with its own structures and logics (Bennett 2017; Al‐Janabi et al. 2019).

Involving relatives in practical nursing care situations and giving them tasks such as how to exercise with the patient were expressed by the nurses as fundamental for involvement. Being actively involved helped the relatives feel more confident, needed and included in the provision of care and, thus, was experienced by the nurses as a facilitator for involvement (Cameron et al. 2013; Lehto et al. 2019). Active involvement of relatives referred to identifying specific possible care tasks, both actual and for future situation of the patient care (Lehto et al. 2019). This required a certain stability and continuity in the collaboration between the relatives and the nurses (Cameron et al. 2014; Lefebvre and Levert 2012; Al‐Janabi et al. 2019). Activating the relatives in the nursing care sometimes worked as a response to prevent emotional distress (Cameron et al. 2013; Lehto et al. 2019).

Sustaining relatives' engagement throughout the rehabilitation process required a continuously purposeful and focused nursing approach (Bennett 2017) that was recognised as a facilitator for involvement. Four studies illustrated that taking time to organise weekend passes facilitates the involvement of relatives because it gives them an opportunity to practice caring for the patient in the home environment, without the direct assistance of HCPs (Cameron et al. 2014, 2013; Dreyer et al. 2016; Aadal et al. 2018). This supported the relatives in what to expect and prepare for once the patient was discharged. As a positive result, relatives gained confidence and felt less fearful about the post‐discharge situation (Cameron et al. 2014, 2013; Dreyer et al. 2016; Aadal et al. 2018).

In nine studies, it was highlighted by the nurses that acknowledging relatives and their individual needs is important when it comes to involving the relatives. Thus, meeting the relatives as individuals with their own needs and embracing the vulnerability the relatives were experiencing reflects the nurses' supportive attitudes towards the relatives and, hence, can be seen as facilitators for involvement (Cameron et al. 2013; Lefebvre and Levert 2012; Dreyer et al. 2016; Aadal et al. 2018; Bennett 2017; Lehto et al. 2019; Ramazanu et al. 2020; Solli et al. 2015; Roy et al. 2015). This was because of the nurses' awareness and knowledge that an ABI affects not only the patient, but the whole family, leaving the relatives facing a crisis and at high risk of becoming ill themselves because of the stressful circumstances (Cameron et al. 2013; Lefebvre and Levert 2012; Dreyer et al. 2016; Aadal et al. 2018; Bennett 2017; Lehto et al. 2019; Ramazanu et al. 2020; Solli et al. 2015; Roy et al. 2015). The nurses considered the relatives' knowledge about the patient's everyday life and routines before the disease important when performing structured and coordinated rehabilitation (Dreyer et al. 2016). The nurses were aware that involving relatives in the patients' care enabled them to observe and address the relatives' needs in the stressful situation, accommodating their vulnerability by showing compassion and support for the relatives (Cameron et al. 2013; Dreyer et al. 2016; Aadal et al. 2018; Solli et al. 2015). The nurses expressed that a core set of values combined with knowledge urged them to purposefully prioritise the involvement of patients and relatives (Bennett 2017). The nurses also emphasised that it was essential that relatives were recognised as persons whose lives were changed forever and not seen as an attachment to the patient, which positively contributed to the involvement of relatives (Aadal et al. 2018). Therefore, a facilitator for involvement was how nurses actively asked the relatives about their previous experiences in coping with crisis and strategically used this information in the involvement (Aadal et al. 2018).

Another pivotal finding was the importance of building a trusting relationship between nurses and relatives as fundamental to involvement (Lefebvre and Levert 2012; Dreyer et al. 2016; Bennett 2017; Lehto et al. 2019; Schutz et al. 2017; Solli et al. 2015; Roy et al. 2015). To be present, to listen to and provide emotional support to the relatives were crucial for relatives to become involved (Schutz et al. 2017; Solli et al. 2015). Listening reflectively and with sensitivity to family members' reactions to the situation helped build a therapeutic relationship and even set the agenda of creating a culture of care for the patient, as well as for the relatives (Bennett 2017; Lehto et al. 2019; Schutz et al. 2017). The nurses articulated that, especially in the first stage of recovery, it was important to invest time and effort to get to know both the patient and the relatives and, accordingly, create a trusting environment that encouraged comfort and open dialogue (Lefebvre and Levert 2012; Dreyer et al. 2016; Bennett 2017).

Furthermore, some nurses expressed almost becoming friends with the family because gaining their trust and confidence was mutually rewarding and enhanced the process of involvement (Bennett 2017). The nurses underlined that, once a trusting relationship was built, it had to be sustained to continuously recognise and respond to the emotional stress the relatives could experience. Helping relatives feel more at ease and meeting the relatives with an empathic, caring and supportive attitude had a positive impact on their involvement (Bennett 2017; Lehto et al. 2019; Schutz et al. 2017; Solli et al. 2015). In one study, the nurses emphasised that they had a role in fostering and sustaining hope for the future, and this was of great importance for the relatives, thus enhancing involvement (Schutz et al. 2017). In addition, when the nurses felt useful in their profession and comfortable in their role as nurses, it became easier for them to interact with and involve the relatives (Bennett 2017; Lehto et al. 2019; Schutz et al. 2017; Roy et al. 2015).

In four studies, communication was discussed as a key tool to involve relatives (Lefebvre and Levert 2012; Aadal et al. 2018; Lehto et al. 2019; Solli et al. 2015). Communication comprised a professional capability to communicate respectfully and emphatically, ask questions, collect data on previous experiences, listen, show sympathy and understand the situation (Lefebvre and Levert 2012; Aadal et al. 2018). Having the time and knowledge specifically on effective communication methods provided the nurses with the flexibility to involve relatives (Solli et al. 2015). Further, having the ability to assess and adjust information individually to relatives and accommodate their personal needs was seen as facilitating involvement (Cameron et al. 2013; Aadal et al. 2018; Al‐Janabi et al. 2019; Kable et al. 2019; Solli et al. 2015; Roy et al. 2015).

### 3.5 Barriers for Involving Relatives From Nurses' Perspective

The most prominent finding occurred in 13 out of the 18 studies, where nurses stressed that the organisational factors of the healthcare system had a negative impact on the possibility to involve relatives (Cameron et al. 2014, 2013; Rochette et al. 2014; Dreyer et al. 2016; Aadal et al. 2018; Kyle et al. 2020; Al‐Janabi et al. 2019; Clancy et al. 2020; Kable et al. 2019; Theofanidis and Gibbon 2016; Ramazanu et al. 2020; Hoewing 2017; Roy et al. 2015). The healthcare system was described in terms of a complex organisation, with its own structures and routines, for example, the ward environment itself being noisy and fast‐paced, interrupted and inconsistent care caused by changing staff (Clancy et al. 2020) or missed appointments and waiting time (Al‐Janabi et al. 2019). The organisation was mostly unfamiliar for the relatives and challenged nurses to consistently involve them (Al‐Janabi et al. 2019; Clancy et al. 2020; Kable et al. 2019). The nurses reported that these organisational time structures challenged the relatives to participate in rounds and team meetings. If the relatives had participated in the meetings concerning the patients, this could potentially have increased the nurses' knowledge about the patient and relatives and enhanced the involving the relatives in the future care (Cameron et al. 2014; Aadal et al. 2018; Kable et al. 2019; Hoewing 2017). The hinders for participating in the meetings were the relatives' private life obligations, such as jobs and taking care of other family members at home (Rochette et al. 2014). Furthermore, the structure of the healthcare system hugely influenced the nurses' ability to provide good communication and personalised care (Kyle et al. 2020). This could, for example, be because of staffing and time constraints and the poor transfer of information between staff during the shift (Clancy et al. 2020). The nurses stated that time was an overall restricting factor to meet the relatives' needs and, hence, perceived as a major barrier towards involvement (Cameron et al. 2014; Dreyer et al. 2016; Aadal et al. 2018; Hoewing 2017; Roy et al. 2015). Combined, restricted time and the relatives' care needs can be perceived as another stressful task for the nurses (Aadal et al. 2018). Nursing staffing shortages and high caseloads on the wards were highlighted as a key resource limitation (Cameron et al. 2013; Theofanidis and Gibbon 2016; Ramazanu et al. 2020), with heavy workloads making it challenging for nurses to create an inviting room for involvement (Aadal et al. 2018).

Preparation in relation to weekend leave or discharge also challenged the nurse's perceived possibility of involving relatives (Cameron et al. 2014, 2013; Kable et al. 2019; Roy et al. 2015). A high caseload combined with last‐minute decisions on sending a patient on weekend leave left the nurses without time to prepare the relatives properly, potentially compromising patient safety (Cameron et al. 2014; Kable et al. 2019).

Relatives being regarded as noncollaborative were described in six of the studies (Cameron et al. 2014; Aadal et al. 2018; Clancy et al. 2020; Theofanidis and Gibbon 2016; Hoewing 2017; Solli et al. 2015) as a direct barrier to involvement. Nurses described how some relatives worked against the nurses in the sense that the relatives felt that they knew the patient better (Clancy et al. 2020) or directly disrupted aspects of the patient's therapy (Theofanidis and Gibbon 2016). Nurses expressed that not all relatives wanted to be involved in rehabilitation because of different perceptions of recovery between relatives and nurses (Aadal et al. 2018; Ramazanu et al. 2020). Nurses perceived it as challenging to engage and involve relatives when they were not consistently present at the hospital. For some relatives, it was a necessary choice to prioritise work over caring for the patient to support their family financially, and in other cases, it appeared that it was because of minimal flexibility from workplaces (Cameron et al. 2014; Ramazanu et al. 2020). The nurses suggested that the current model of planning training and meetings should be scheduled to better accommodate relatives' work hours (Cameron et al. 2014).

In general, the nurses considered the patient as their primary focus of care and sometimes found it challenging and filled with dilemmas to involve relatives in stroke care and decision‐making (Rochette et al. 2014; Aadal et al. 2018; Kable et al. 2019). On the one hand, nurses considered relatives as a resource (Aadal et al. 2018), but on the other hand, relatives were recognised as a stressful task because of the time restrictions that nurses work within (Cameron et al. 2013; Rochette et al. 2014; Dreyer et al. 2016; Aadal et al. 2018; Kable et al. 2019; Roy et al. 2015). Even though the nurses were aware that the relatives were in crisis and acknowledged that they were in a difficult situation, the nurses perceived a lack of time and resources to plan and adjust their care to include the caring for and involvement of the relatives (Dreyer et al. 2016; Aadal et al. 2018; Roy et al. 2015). The nurses expressed that they perceived themselves as having a dual role in relation to both getting the relatives to cooperate regarding the patient while caring for the relatives (Aadal et al. 2018).

A total of two studies pointed out that there is a lack of knowledge and tools for identifying patients' and relatives' informational needs at different stages of their disease trajectory and that these were highlighted as desired (Kyle et al. 2020; Hoewing 2017). The relatives' individual readiness to receive information was perceived by the nurses as a barrier to involvement (Cameron et al. 2014, 2013; Lefebvre and Levert 2012; Aadal et al. 2018; Bennett 2017; Kable et al. 2019). Even though the nurses were aware that the relatives needed individually tailored information, they expressed a struggle to determine when relatives were ready to receive information and often felt that information could have been provided earlier (Cameron et al. 2014, 2013; Lefebvre and Levert 2012; Bennett 2017; Kable et al. 2019; Theofanidis and Gibbon 2016; Hoewing 2017; Roy et al. 2015). The different perceptions of recovery were further expressed by limited conversations about the care decisions between the patient, relatives and nurse and, hence, were perceived as a barrier towards the effective coordination of care by nurses to both the patients and their relatives (Ramazanu et al. 2020).

Three studies furthermore explicitly described language as a barrier to involving relatives, literally in situations where nurses, patients and relatives had different native languages (Lefebvre and Levert 2012; Ramazanu et al. 2020; Roy et al. 2015). A facilitator in these situations were visual aids and translated pamphlets that were suggested to accommodate this barrier. Furthermore, healthcare jargon or professional healthcare language was experienced as a barrier to mutual understanding (Roy et al. 2015).

## 4 Discussion

The objective of the present scoping review was to identify and map the breadth of available evidence on the possible facilitators and barriers to nurses' involvement of relatives of patients with ABI and MBT. Surprisingly, no studies were found focusing specifically on the MBT population, indicating a large literature gap in this population. Although the findings are particularly focused on the relatives of patients with ABI the results may be generalised to the relatives of other patient populations as well. This broader relevance arises from the widespread recognition within healthcare systems of the importance of engaging relatives in patient care and treatment (Lundh et al. 2024).

Eighteen papers describing the different aspects of perceived facilitators and barriers were included, reflecting the fact that the research related to this topic is still rare yet has increased over the past decade. Most studies were published in Europe and North America, underscoring the increasing emphasis in the Western world on viewing the involvement of relatives in patient care as essential when considering the individual patient's life circumstances in planning and delivering care (Jørgensen et al. 2022). This trend can likely be attributed to international bodies calling for increased patient and family involvement and reflects a broader societal acknowledgement of relatives' significance in healthcare (Olding et al. 2016).

In 7 out of the 18 studies, the demographic information of the participating nurses and HCPs was reported in limited detail and in varying ways. This could be a possible result of only including studies on the relatives of patients with ABI. In most stroke rehabilitation, nurses are part of multidisciplinary team, and most of the included studies did not focus on the specific professions, but rather on the entire team as hole. The number of nurses and different groups of HCPs demonstrates that the care trajectory is characterised by collaboration between several HCPs. However, no conclusion can be drawn regarding the implications of interprofessional collaboration between HCPs. Nevertheless, Hetland et al. found that nurses are more likely than other HCPs to involve family members in patient care and that age, education, work experience, specialisation and staffing ratios influence the involvement of relatives (Hetland et al. 2017). This trend resonates with the pervasive role expectation that nurses play a leading role in promoting and facilitating patient and relative involvement (Olding et al. 2016).

The characteristics of the relative population in the included studies revealed that 68% represents close family members, distributed on 34% spouses/partners to the patients, 17% having a parental relation, whereas 16% were adult children of the patients. The remaining relatives represents other family relations or other relations. This finding highlights the need for future research to focus on the nature of the relationship between the patient and closest relative, especially considering the ongoing trend of an expanding number of adults living on their own (Our World in Data 2019).

The findings of the scoping review indicate that the nurses' ability to facilitate relatives' involvement throughout the patient care trajectory is complicated and influenced by different aspects. We propose that these facilitators and barriers to involvement are not mutually exclusive but rather represent aspects of involvement that range along a continuum from (1) facilitators and barriers related to the healthcare system, including organisational factors and to (2) facilitators and barriers related to the psychosocial, relational, physical needs of the relatives, including communication, information, being respected and listened to.

The complexity of the organisational factors necessitates nurses to assist relatives in navigating the health care system, enhancing their understanding of the system, and demonstrating the ways they can participate in the patient's care. However, the wider processual, organisational and contextual structure and limited resources that shape the conditions for involving relatives within the healthcare system consistently emerge as the most prominent barriers. This opposing factor is also found in a literature‐based study underscoring that there are institutional, communicational, environmental and personal/behavioural–related barriers that influence nurses' involvement of relatives within the healthcare context (Kwame and Petrucka 2021). Furthermore, the findings emphasise that effective organisation of the workflow plays a crucial role in the nurses' ability to easing the care trajectory for the patients and relatives, for example, securing continuity, interprofessional collaboration and planning transitions between the different healthcare sectors, that is, primary and secondary healthcare. Addressing these challenges calls for smart organisational planning and strategies, taking into consideration that the World Health Organization (WHO) has projected a global shortage of approximately 50% in the nursing workforce, which further underscores the need for innovative approaches and solutions (World Health Organization 2020).

The facilitators and barriers for involvement were found to be related to psychosocial, relational and physical needs, including communication, information, respect and being listened to. One of these findings illustrate how the nurses struggled to meet relatives with their own needs. This could be because relatives are viewed as vulnerable subjects that must be brought into the fold of care as resources for improving patient outcomes but not as individuals to be partnered with by HCPs in the care of the patient (Olding et al. 2016).

Another finding was that the establishment of a trusting relationship between the relatives and nurse was crucial for the relatives to be involved. Guldager et al. (2023) and Guldager, Nordentoft, et al. (2022) Guldager et al. (2019) found that focusing on building trusting relationships between relatives to patients with ABI or MBT and nurses, along with meeting the relatives' need for information, may enhance the involvement of relatives and even increase the relatives' confidence in their caregiver role and sense of control over the situation. Building on the framework of Fundamentals of Care, Kitson (Kitson et al. 2013) states that the focus of care is the patient, however, relatives are also mentioned as having own needs for involvement and support. Yet, also emphasises that patients with cognitive impairments and/or communication difficulties following, for example, ABI place a greater demand on the nurses and their need to involve and care for relatives, to achieve the integration of care (Kitson et al. 2013). This underlines the importance of conceptualising relatives and patients as a family unit to be taking care of, not least to determine how the relatives see themselves as integral to the treatment in the care trajectory (Creasy et al. 2015).

### 4.1 Nurses Involvement in the Research Process

A panel of five nurses from neurosurgery semi‐intensive care unit (*n* = 2), bed ward (*n* = 2) and outpatient clinics (*n* = 1) were involved in reviewing the results prior to submitting this scoping review. This panel of nurses could easily relate to the findings into a (Danish) context, recognising the importance of involving relatives and viewing them as a valuable resource and experts in the patients' lives. They also found it straightforward to relate to the organisational barriers linked to the specific context in which involvement should take place. Furthermore, the nurses contributed some new perspectives, primarily pertaining to cultural factors and the significance of biological relations concerning involvement (e.g., regarding the designation of the primary relative when a patient is unconscious). These new perspectives will be applied in an upcoming research project.

### 4.2 Implication for Practice and Research

This scoping review highlights that nurses have a central role in nurturing a trustful relationship with relatives, which is crucial as a fundament for involvement. Further those socio‐cultural processes, including organisational and contextual factors, shape relative involvement. The review shed lights on the importance of relational dynamics in involvement and underscores a diverse spectrum within the relative population, coinciding with projections of an increasing number of individuals living independently in the future. Future research should focus on nurses' facilitators and barriers towards involvement of relatives in the MBT population because there is a gap in the literature regarding patients diagnosed with MBT. In addition, future research is needed to better conceptualise ‘involvement’ to achieve consensus and clarify the meanings and differences. This is because the conceptual ambiguity might influence how nurses perceive and assess involvement in practice and, thus, perceive facilitators and barriers to involvement.

### 4.3 Limitations of the Review

The strengths of this scoping review include the development and publication of an a priori protocol (Guldager, Loft, et al. 2022), however, we chose to explore involvement of relatives in general and not only in nursing care decisions or selfcare. The focus for the review was relatives of patients with ABI or MBT, and no studies were found specifically focusing on the MBT population. Although we followed a structured framework and used a comprehensive search strategy, it is possible that relevant studies were missed. It could be the case if some studies included a broader population of patients with a cancer diagnosis, including patients with MBT. A limitation of the review may be the date filter restriction. However, the context for the involvement of relatives in patient care has changed markedly over the past decade and thus the restriction makes our review more relevant. Several factors have contributed to these changes, for example, a growing elderly population, introduction of fast‐track programmes and shortened lengths of hospital stay (Norlyk and Martinsen 2013).

## 5 Conclusion

In this scoping review, we have mapped the available evidence from the international literature regarding nurses' facilitators and barriers related to involving relatives in all aspects of the care continuum of patients with ABI. No studies including involvement of relatives to patients with MBT was found. The results illustrate the comprehensive and complex nature of involvement. Our results indicate that there are several aspects that are not mutually exclusive but rather represent aspects of involvement that range along a continuum from (1) facilitators and barriers related to the healthcare system, including organisational factors in terms of resources to (2) facilitators and barriers related to the psychosocial, relational, physical needs of the relatives. These needs include communication, information, being respected and listened to and building a relationship, which are pivotal to successful involvement of relatives in the patient care continuum. We suggest that the organisational and contextual factors that shape relative involvement and involvement of relatives to patients with MBT need to be studied further and that strengthening the relationships between relatives and nurses is needed to individualise and optimise the level of involvement.

## Author Contributions

R.G., L.A., M.L., I.P. contributed to the study design. R.G. and I.P. contributed to the development of the eligibility criteria. R.G., S.N. and P.S.S. contributed to the data extraction criteria and the search strategy. All authors contributed to the review approach design, data analysis and writing manuscript.

## Funding

This work was supported by Danish Health Confederation and Danish Regions, 2657.

## Disclosure

The present study forms part of a larger research project registered at ClinicalTrials.gov (‘Development and Implementation of an Intervention Enhancing Involvement of Relatives to Patients With Acquired Brain Injury or Malignant Brain Tumour’, ClinicalTrials.gov Identifier: NCT06796335).

## Ethics Statement

The authors have nothing to report.

## Conflicts of Interest

The authors declare no conflicts of interest.

## Supporting information


**Data S1:** nop270417‐sup‐0001‐DataS1.docx.

**Appendix S1–S4:** nop270417‐sup‐0002‐AppendixS1‐S4.docx.

## Data Availability

The data that support the findings of this study are available from the corresponding author upon reasonable request.
