# Supplementary material for: Facilitators and Barriers of Relatives' Involvement in Care of Patients With Acquired Brain Injury or Malignant Brain Tumour: Scoping Review
Source: Nurs Open. 2026 Jan 20;13(1):e70417. doi: 10.1002/nop2.70417 (PMC12819177; doi:10.1002/nop2.70417)
Supplement: Supplementary file 2 — Appendix S1–S4: nop270417‐sup‐0002‐AppendixS1‐S4.docx. [file NOP2-13-e70417-s002.docx]

### **Appendix I: Search strategy**

Initial search strategy for MEDLINE (PubMed)

December 2021: researched, updated, and rerun in October 2022 and December 2023.

| **Search ID#** | **Search Terms** | **Results** |
| --- | --- | --- |
| S15 | 14 and (2022* or 2023*).rd. | 230 |
| S14 | 3 and 7 and 10 and 13 | 730 |
| S13 | 11 or 12 | 798,224 |
| S12 | exp Brain Injuries/ or exp Craniocerebral Trauma/ or Cerebrovascular Trauma/ or exp Stroke/ or exp Brain Neoplasms/ or exp Glioma/ | 556,401 |
| S11 | ("brain injur*" or stroke or "brain neoplasm*" or "brain cancer*" or glioma* or glioblastoma*).ti,ab,kf. | 510,181 |
| S10 | 8 or 9 | 455,1889 |
| S9 | Professional-Family Relations/ or exp Family/ or Caregivers/ or Family Nursing/ | 417,038 |
| S8 | (family or families or relative* or caregiver* or son or sons or daughter* or child* or spouse* or mother* or father*).ti,ab,kf. | 443,4305 |
| S7 | 4 or 5 or 6 | 370,7527 |
| S6 | Decision Making, Shared/ or Needs Assessment | 34,549 |
| S5 | ((family adj3 need) or (family adj3 needs)).ti,ab,kf. | 6,184 |
| S4 | (involv* or engag* or participat* or collaborat*).ti,ab,kf. | 3,677,094 |
| S3 | 1 or 2 | 7143,56 |
| S2 | (nurse or nurses or nursing).ti,ab,kf. | 528,637 |
| S1 | exp Nurses/ or exp Nursing Staff/ or exp Nursing/ or exp Nursing Care/ or Neuroscience Nursing/ | 415,108 |

## Initial search strategy for EBSCOhost (via Cinahl)

December 2021: researched, updated, and rerun in October 2022 and December 2023.

| **Search ID#** | **Search Terms** | **Results** |
| --- | --- | --- |
| S15 | S3 AND S7 AND S10 AND S13 | 28 |
| S14 | S3 AND S7 AND S10 AND S13 | 603 |
| S13 | S11 OR S12 | 251,064 |
| S12 | (MH "Brain Injuries+") OR (MH "Head Injuries+") OR (MH "Cerebrovascular Disorders+") OR (MH "Stroke+") OR (MH "Brain Neoplasms+") OR (MH "Glioma+") | 194,657 |
| S11 | TI "brain injur*" OR TI stroke OR TI "brain neoplasm*" OR TI "brain cancer*" OR TI glioma* OR TI glioblastoma* OR AB "brain injur*" OR AB stroke OR AB "brain neoplasm*" OR AB "brain cancer*" OR AB glioma* OR AB glioblastoma* | 156,626 |
| S10 | S8 OR S9 | 1,222,988 |
| S9 | (MH "Professional-Family Relations") OR (MH "Family Nursing") OR (MH "Caregivers") OR (MH "Family+") | 314,721 |
| S8 | TI family OR TI families OR TI relative* OR TI caregiver* OR TI son OR TI sons OR TI daughter* OR TI child* OR TI spouse* OR TI mother* OR TI father* OR AB family OR AB families OR AB relative* OR AB caregiver* OR AB son OR AB sons OR AB daughter* OR AB child* OR AB spouse* OR AB mother* OR AB father* | 1,120,666 |
| S7 | S4 OR S5 OR S6 | 813,218 |
| S6 | (MH "Decision Making, Shared") OR (MH "Needs Assessment") | 26,833 |
| S5 | TI (family N3 need) OR TI (family N3 needs) OR AB (family N3 need) OR AB (family N3 needs) | 9,653 |
| S4 | TI involv* OR TI engag* OR TI participat* OR TI collabor* OR AB involv* OR AB engag* OR AB participat* OR AB collabor* | 787,431 |
| S3 | S1 OR S2 | 832,895 |
| S2 | TI nurse OR TI nurses OR TI nursing OR AB nurse OR AB nurses OR AB nursing | 614,153 |
| S1 | (MH "Neuroscience Nursing+") OR (MH "Neuroscience Nurses") OR (MH "Nursing Care+") OR (MH "Staff Nurses") OR (MH "Nursing Staff, Hospital+") OR (MH "Nursing Home Personnel") OR (MH "Nurses+") | 518,509 |

## Initial search strategy for Ovid Advanced Searching (via Embase)

December 2021: researched, updated, and rerun in October 2022 and December 2023.

| **Search ID#** | **Search Terms** | **Results** |
| --- | --- | --- |
| S15 | 14 and (2022* or 2023*).dc,rd. | 282 |
| S14 | 3 and 7 and 10 and 13 | 2,102 |
| S13 | 11 or 12 | 1,298,911 |
| S12 | exp brain injury/ or exp head injury/ or exp traumatic brain injury/ or exp cerebrovascular accident/ or exp brain tumor/ or exp glioma/ or exp glioblastoma/ | 996,116 |
| S11 | ("brain injur*" or stroke or "brain neoplasm*" or "brain cancer*" or glioma or glioblastoma*).ti,ab,kf. | 765,978 |
| S10 | 8 or 9 | 6,408,463 |
| S9 | exp human relation/ or exp family/ or exp nuclear family/ or exp caregiver/ or family nursing/ or exp family centered care/ | 1,501,187 |
| S8 | (family or families or relative* or caregiver* or son or sons or daughter* or child* or spouse* or mother* or father*).ti,ab,kf. | 5559141 |
| S7 | 4 or 5 or 6 | 4,750,021 |
| S6 | exp shared decision making/ or exp family decision making/ or needs assessment/ | 45,093 |
| S5 | ((family adj3 need) or (family adj3 needs)).ti,ab,kf. | 7,656 |
| S4 | (involv* or engag* or participat* or collaborat*).ti,ab,kf. | 4,713,821 |
| S3 | 1 or 2 | 833,730 |
| S2 | (nurse or nurses or nursing).ti,ab,kf. | 610,797 |
| S1 | exp nurse/ or exp nursing staff/ or exp nursing/ or exp nursing care/ or exp neuroscience nursing/ or exp nursing practice/ | 601,077 |

Initial search strategy for Web of Science

December 2021: researched, updated, and rerun in October 2022 and December 2023.

| **#** | **Search** | **Hits** |
| --- | --- | --- |
| 1 | acquired brain injury OR stroke OR brain injuries OR glioblastoma AND relatives OR family OR carers OR family relations OR wife OR husband OR partner OR child OR spouse OR sibling OR brother OR sister AND involvement OR family needs OR shared decision making OR relative involvement OR facilitators OR barriers (all fields) | 3,937,894 |
| 2 | acquired brain injury OR stroke OR brain injuries OR glioblastoma AND relatives OR family OR carers OR family relations (all fields) | 1,912,268 |
| 3 | relatives OR family OR carers OR family relations AND involvement OR family needs OR shared decision making OR relative involvement AND facilitators OR barriers (all fields) | 3,096,949 |
| 4 | facilitator AND involvement AND nurses (All fields) | 330 |
| 5 | Barrier AND involvement AND nurses (All fields) | 1,007 |
| 6 | Facilitators OR Barriers AND nurses AND involvement (All fields) | 41,543 |
| 7 | **nursing staff involvement of relatives**(All Fields) | 211 |
| 8 | **Nurses’ barriers or facilitators for involvement of relatives (All fields)** | 17,785 |

Timespan: 2010-01-01 to 2023-01-05 (Publication Date)

Initial search strategy for Google Scholar

December 2021: researched, updated, and rerun in October 2022 and December 2023..

| **#** | **Search (Find articles)** | **Hits** |
| --- | --- | --- |
| 1 | nursing staff involvement of relatives | 19,800 |
| 2 | Barrier involvement | 17,800 |
| 3 | Facilitators involvement | 18,100 |
| 4 | nurse involvement of relatives acquired brain injury | 19,100 |
| 5 | nurse involvement of relative’s brain cancer | 19,500 |
| 6 | nurse involvement of relative’s stroke | 19,000 |
| 7 | facilitators and barriers of nurse involvement of relatives acquired brain injury | 17,800 |
| 8 | facilitators and barriers of nurse involvement of relative’s brain cancer | 17,100 |
| 9 | facilitators and barriers of nurse involvement of relative’s stroke | 17,300 |

Articles dated between 2010 and present

### **Appendix II: List of excluded studies**

Excluded studies (n=39)

| **Author**  **Year of publication** | **Title** | **Reason for exclusion** |
| --- | --- | --- |
| Al-Janabi H. et al.  2019 | Six mechanisms behind carer wellbeing effects: a qualitative study of healthcare delivery | Duplicate |
| Antoniou T.  2018 | Educational preparation and training of the family caregivers of elderly patients with stroke in the pre-discharge period: A proposed innovation | Wrong focus |
| Aoun S. et al.  2015 | Exploring the support needs of family caregivers of patients with brain cancer using the CSNAT: a comparative study with other cancer groups | Wrong perspective |
| Applebaum A.J. et al.  2018 | Prognostic awareness and communication preferences among caregivers of patients with malignant glioma | Wrong perspective |
| Araujo O. et al.  2015 | Intervention in informal caregivers who take care of older people after a stroke (InCARE): study protocol for a randomised trial | Wrong focus |
| Bakas T. et al.  2015 | Telephone assessment and skill-building kit for stroke caregivers: a randomized controlled clinical trial | Wrong focus |
| Bakas T. et al.  2016 | Tracking patterns of needs during a telephone follow-up programme for family caregivers of persons with stroke | Wrong perspective |
| Cameron J. et al.  2013 | Stroke family caregivers' support needs change across the care continuum: a qualitative study using the timing it right framework | Duplicate |
| Cameron J. et al.  2015 | A feasibility and pilot randomized controlled trial of the “Timing it Right Stroke Family Support Program” | Wrong perspective |
| Cecil R. et al.  2013 | Towards an understanding of the lives of families affected by stroke: a qualitative study of home carers | Wrong perspective |
| Chang A. et al.  2015 | A family involvement and patient-tailored health management program in elderly Korean stroke patients' day care centers | Wrong perspective |
| Christiansen B. et al.  2017 | Challenges in the nurse’s role in rehabilitation contexts | Wrong scope |
| Coco K. et al.  2013 | The provision of emotional support to the families of traumatic brain injury patients: perspectives of Finnish nurses | Wrong focus |
| Coco K. et al.  2011 | Support for traumatic brain injury patients' family members in neurosurgical nursing: a systematic review | Wrong perspective |
| Cowey E. et al.  2015 | Impact of a clinical pathway on end-of-life care following stroke: a mixed methods study | Wrong scope |
| Creasy K. et al.  2015 | Clinical implications of family-centered care in stroke rehabilitation | Wrong perspective |
| Farnese M. et al.  2020 | Caregivers' engagement during in-hospital care of sABI's patients: evaluation of informal co-production from the health providers' perspective | Wrong scope |
| Foster A. et al.  2012 | Encouraging family engagement in the rehabilitation process: a rehabilitation provider's development of support strategies for family members of people with traumatic brain injury | Wrong study design |
| Gebhardt M. et al.  2011 | Caregiver and nurse hopes for recovery of patients with acquired brain injury | Wrong outcomes |
| Jones K. et al.  2020 | Rehabilitation health professionals' perceptions of spirituality and spiritual care: the results of an online survey | Wrong scope |
| Kiker W. et al.  2021 | Assessment of discordance between physicians and family members regarding prognosis in patients with severe acute brain injury | Wrong scope |
| Levasseur M. et al.  2016 | Identifying participation needs of people with acquired brain injury in the development of a collective community smart home | Wrong population (no nurses) |
| Magwood G. et al.  2019 | Barriers and facilitators of stroke recovery: perspectives from African Americans with stroke, caregivers and healthcare professionals | Wrong perspective |
| McCurley J. L. et al.  2019 | Preventing chronic emotional distress in stroke survivors and their informal caregivers | Wrong focus |
| Meixner C. et al.  2017 | Impact of the Brain Injury Family Intervention (BIFI) training on rehabilitation providers: a mixed methods study | Wrong perspective |
| Oyesanya T. O. et al.  2021 | “Just tell me in a simple way”: a qualitative study on opportunities to improve the transition from acute hospital care to home from the perspectives of patients with traumatic brain injury, families, and providers | Wrong perspective |
| Philip J. et al.  2015 | Health care professionals’ perspectives of living and dying with primary malignant glioma: Implications for a unique cancer trajectory. | Wrong scope |
| Pierce L. et al.  2015 | Qualitative analysis of a nurse's responses to stroke caregivers on a web-based supportive intervention | Wrong perspective |
| Rochette A. et al.  2013 | Ethical issues relating to the inclusion of relatives as clients in the post-stroke rehabilitation process as perceived by patients, relatives and health professionals | Wrong focus |
| Ryan M. et al.  2014 | How comfortable are clinicians with discussing difficult issues for patients with acute stroke? | Abstract only |
| Souseme G. et al.  2022 | Barriers and facilitators linked to discharge destination following inpatient rehabilitation after traumatic brain injury in older adults: a qualitative study | Wrong population (no nurses) |
| Sousme G. et al  2020 | Returning home after inpatient rehabilitation for geriatric traumatic brain injury: facilitators and barriers identified by patients, family members, and healthcare professionals | Only abstract |
| Spetz A. et al.  2005 | A specialist nurse-function in neurooncology: a qualitative study of possibilities, limitations, and pitfalls | Wrong perspective |
| Stork R. et al.  2018 | The family caregiving dilemma | Wrong focus |
| Sundin K. et al.  2018 | Envisioning the future as expressed within family health conversations by families of persons suffering from stroke | Wrong scope |
| Sutton K. M. et al.  2019 | Engaging individuals with neurological conditions and caregivers in rural communities in a health research team | Wrong population (no nurses) |
| Villanueva N. E.  1999 | Experiences of critical care nurses caring for unresponsive patients | Published before 2010 |
| Yetman L.  2009 | Caring for families: Double binds in neuroscience nursing | Published before 2010 |
| Wu C. Z. et al.  2022 | Perceived challenges in delivering comprehensive care for patients following stroke: a qualitative study of stroke care providers in Guangdong Province, China. | Wrong scope |

### **Appendix III: Data extraction tool**

| Study ID | - Author - Year of publication - Country of origin |
| --- | --- |
| Methodology/methods | - Qualitative studies - Quantitative studies - Mixed methods or multimethods |
| Qualitative study design | - Individual interviews - Focus group interviews - Observational studies |
| Quantitative study design | - Randomised controlled trail - Pilot randomised controlled trail - Quasi-experimental - Cohort studies - Case control - Other |
| Mixed or multimethods | - Interview and questionnaires - Interview and observations - Interview and randomised controlled trail |
| Type of intervention | - Educational - Emotional/cognitive - Informational - Psychosocial - Physical |
| Modes of delivery | - In person (individual, groups) - Online - Telephone |
| Intervention provided by | - Nurses - Therapists, including physio-, occupational and speech therapists - Doctors, including neurologists, neurosurgeons and oncologists - Neuropsychologists |
| Data collection | - Cross-sectional (one data point) - Longitudinal (two or more data points) |
| Setting | - Hospital - In- or outpatient - Community based - At home |
| Study population – ABI | - ABI - TBI - Stroke |
| Study population – MBT | - Primary brain tumour - High-grade glioma |
| Duration of intervention | - 1–2 weeks - 2 weeks–3 months - 3 months–6 months - 6 months–12 months |
| Outcome/measures | - Qualitative - Quantitative - Qualitative and quantitative |
| Evaluation of the intervention | - Yes - No |

**Appendix IV: Characteristics of the included studies**

| **Title** | **Author/ Year of public-cation/ Country of origin** | | **Aims** | | **Study population and sample size** | | **Relatives** | | **Patients’ diagnosis** | | **Recruited/**  **Setting/context** | | **Methods** | | **Design** | |
| --- | --- | --- | --- | --- | --- | --- | --- | --- | --- | --- | --- | --- | --- | --- | --- | --- |
| Six mechanisms behind carer wellbeing effects: A qualitative study of healthcare delivery | Al-Janabi, H. et al.  2019  UK | | To empirically determine mechanisms by which health and social care services affected family careers lives | | 25 care professionals, including 8 doctors/nurses | | N= 24  9 parents  9 spouses  5 children | | Dementia  Stroke  Mental health | | Charity organisations in the three clinical areas + network | | Focus group interviews | | A qualitative study | |
| A culture of caring: how nurses promote emotional wellbeing and aid recovery following a stroke | Bennett B.  2017  UK | | To explore patients’ emotional experiences following a stroke during hospital-based rehabilitation and to examine how nurses use their knowledge to inform their interactions with patients and families | | 14 nurses and 17 healthcare assistants | | N =10  3 spouses  3 children  4 not reported | | Stroke | | Acute and rehabilitation phase | | Participant observation, interviews and document review | | A qualitative study | |
| Stroke family caregivers' support needs change across the care continuum: a qualitative study using the timing it right framework | Cameron J. et al.  2013  Canada | | To: (1) explore the support needs over time from the perspective  of caregivers, (2) explore the support needs over time from the  perspective of HCPs, and (3) compare and contrast caregivers’ and HCPs’ perspectives | | 14 HCP, including 5 nurses | | N= 24  18 spouses  6 children | | Stroke | | Acute care postdischarge phase | | In-depth interviews | | A qualitative study | |
| Stroke survivors', caregivers', and health care professionals' perspectives on the weekend pass to facilitate transition home | Cameron J. et al.  2014  Canada | | To explore stroke survivors’, caregivers’, and health care professionals’ perceptions of weekend passes offered during inpatient rehabilitation and its role in facilitating the transition home | | 20 HCP, including 3 nurses | | N= 15  10 parents  3 spouses  2 children | | Stroke | | In-patient rehabilitation hospital | | Focus groups and in-depth interviews | | A qualitative study | |
| “Living in a foreign country”: Experiences of staff-patient communication in inpatient stroke settings for people with post stroke aphasia and those supporting them | Clancy, L. et al.  2020  UK | | To explore staff-patient communication in in-patient stroke settings for stroke survivors with aphasia and those supporting them, from the perspectives of strokes survivors, their carers and healthcare professionals | | 6 HCP incl. 1 nurse | | N= 10  8 spouses  2 children | | Stroke | | National health service hospital sites | | Interviews and focus group interviews | | A qualitative study | |
| Nursing roles and functions in the acute and subacute rehabilitation of patients with stroke: Going all in for the patient | Dreyer, P. et al.  2016  Denmark | To describe the experienced roles and functions of nurses during in-hospital rehabilitation of patients with stroke | | 19 nurses | | N= 0 | | Stroke | | Acute care setting, highly specialised rehabilitation ward and rehabilitation ward | | Focus group interviews | | A qualitative study | |  |
| Assessing the quality of education and information delivery to family members of patients with moderate to severe traumatic brain injury | Hoewing, B.  2021  USA | To perform a unit-based needs assessment of quality of education and information delivery given by nurses and providers to family members of patients with moderate to severe TBI.  Analyse survey results to determine themes and topics to be included in TBI family education for the ICU  Use survey results to develop and recommend a standardised education program to be delivered to family members of patients with TBI | | ICU staff members incl. 17 nurses | | N= 5  3 parents  2 children | | Moderate to severe TBI | | ICU | | Interviews  Surveys, quantitative questions with open ended questions | | Mixed methods | |  |
| Health professionals' perspectives on the discharge process and continuity of care for stroke survivors discharged home in regional Australia: A qualitative, descriptive study | Kable A. et al.  2019  Australia | To understand health professionals’ perspectives on the discharge process and continuity of care during the transition between hospital and home for stroke survivors | | 25 HCP, including 15 nurses | | N= 0 | | Stroke | | In-patient rehabilitation | | Focus group interview | | A qualitative study | |  |
| What helps and hinders the provision of healthcare that minimizes treatment burden and maximizes patient capacity? A qualitative study of stroke health professional perspectives | Kyle J. et al.  2020  UK | To examine the potential barriers and enablers to minimising treatment burden and maximising patient capacity faced by health professionals and managers providing care to those affected by stroke | | 21 HCP, including 3 nurses | | N= 0 | | Stroke | | Primary and secondary care stroke services | | Semistructured interviews | | A qualitative study | |  |
| The needs experiences by individuals and their loved ones following a traumatic brain injury | Lefebre, H. & Levert, M.J.  2012  France/Canada | To explore the needs of individuals and their loved ones throughout the continuum of care and services, from the point of view of everyone affected by the experience of a TBI, including individuals, their loved ones, and the health care professionals involved in their care | | 60 HCP including 5 nurses | | N= 34  Not reported | | TBI | | Staff:  Acute care, rehabilitation, social integration | | Focused discussion groups guided by a questionnaire | | A qualitative explorative design | |  |
| Caring Interaction with stroke survivors' family members-Family members' and nurses' perspectives | Lehto B. et al.  2019  Finland | To examine emotional support given by nurses to family members in the acute phase after a working‐aged patient's stroke based on nurses’ and family members’ experiences | | 12 nurses | | N= 17  Not reported | | Stroke | | Acute and rehabilitation phase | | Focus group interviews | | A qualitative study | |  |
| The experiences of couples affected by stroke and nurses managing patient rehabilitation: a descriptive study in Singapore | Ramazanu, S. et al.  2020  Singapore | To explore the experiences of stroke from the perspectives of couples affected by stroke and the nurses managing patient rehabilitation | | 8 nurses | | N= 5  5 spouses | | Stroke | | Rehabilitation phase | | Individual, in-depth interviews | | A qualitative study | |  |
| Actual and ideal services in acute care and rehabilitation for relatives’ post-stroke from three perspectives: relatives, stroke clients and health professionals | Rochette A. et al.  2014  Canada | To explore actual services received by relatives and to contrast it to desired ideal services (i.e., services wished for relatives by participants) in acute care and poststroke rehabilitation from 3 perspectives: relatives, stroke clients and health professionals | | 1 nurse | | N= 25  1 parent  8 spouses  2 siblings  9 children  3 other family  2 not family | | Stroke | | Acute care, in-patient or outpatient rehabilitation | | In-depth interviews and focus groups | | A qualitative study | |  |
| Health professionals and family perceptions of post-stroke information | Roy, et al.  2015  New Zealand | To ascertain information and education needs of families of those who experience a stroke (stroke survivors) through identifying current practice and resources, and the appropriateness, accessibility, method of delivery, timeliness and gaps in education and information giving | | 23 HCP including 5 nurses | | N = 19  10 parents  1 sibling  7 children | | Stroke | | Acute, rehabilitation | | Survey  interview | | Mixed methods | |  |
| Is there hope? Is she there? How families and clinicians experience severe acute brain injury | Schutz, BA. et al.  2017  USA | To explore how family members, nurses and physicians experience the palliative and supportive care needs of patients with SABI receiving care in the neuroscience intensive care unit (neuro-ICU) | | 15 nurses  16 physicians | |  | | SBI | | Neuro-ICU | | Semistructured interviews | | A qualitative study | |  |
| Characteristics of the relationship that develops from nurse-caregiver communication during telecare | Solli, H. et al.  2015  Norway | To explore the relationship between nurses and caregivers using a web camera forum as the communication methods | | 4 nurses  2 nurse-assistants | | N= 9  9 spouses | | Stroke  Dementia | | Public health care | | Semistructured interviews | | A qualitative study | |  |
| Exploring the experiences of nurses and doctors involved in stroke care: a qualitative study | Theofandis D.& Gibbon B.  2016¨  Greece | To describe the current stroke care delivery arrangements in Greece and explore nursing and medical staff knowledge, skills and attitudes towards stroke care and management | | 6 medical staff  15 nursing staff | |  | | Stroke | | Acute and rehabilitation phase | | Semistructured interviews | | A qualitative study | |  |
| Nursing roles and functions addressing relatives during in-hospital rehabilitation following stroke. Care needs and involvement | Aadal L. et al.  2018  Denmark | To describe nurses’ experienced roles and functions addressing the relatives of patients with stroke during in-hospital rehabilitation | | 19 nurses | |  | | Stroke | | Acute care setting, highly specialised rehabilitation ward and rehabilitation ward | | Focus group interviews | | A qualitative study | |  |

Abbreviations

HCP: Healthcare professionals

SBI: Severe acute brain injury

TBI: Traumatic brain injury
